# Use of vasopressor for dialysis-related hypotension is a risk factor for death in hemodialysis patients: Nationwide cohort study

**DOI:** 10.1038/s41598-019-39908-6

**Published:** 2019-03-04

**Authors:** Eiichiro Kanda, Yuki Tsuruta, Kan Kikuchi, Ikuto Masakane

**Affiliations:** 1 Medical Science, Kawasaki Medical School, Okayama, Japan; 2 Tsuruta Itabashi Clinic, Tokyo, Japan; 3 Shimoochiai Clinic, Tokyo, Japan; 4 Department of Nephrology, Honcho Yabuki Clinic, Yamagata, Japan

## Abstract

Because hypotension during hemodialysis (HD) makes continuation of HD difficult and is associated with mortality, pressor approaches are necessary for patients with hypotension. However, the relationships between the pressor approaches and the risk of death have not been clarified yet. We analyzed data from a nationwide prospective cohort study of the Japanese Society for Dialysis Therapy Renal Data Registry (n = 29,309). The outcome was all-cause one-year death. The association between the use of pressor approaches and the outcome was examined using Cox proportional hazards models adjusted for baseline characteristics, propensity score matched analysis and Bayesian networks. The background features of the patients were as follows: male, 59.6%; average age, 64.5 ± 12.5 years; and patients with diabetes mellitus, 31.5%. The pressor group showed a higher risk of the outcome than the control group [adjusted hazard ratio (aHR) 1.33 (95% CI: 1.21, 1.47), *p* = 0.0001]. Propensity score matched analysis also showed that the matched-pressor group had a higher risk of the outcome than the matched-control group [aHR 1.30 (95% CI: 1.17, 1.45), *p* = 0.0001]. Moreover, the Bayesian network showed a direct causal relationship from the use of pressor approaches to the outcome. The use of oral vasopressors [aHR 1.20 (95% CI: 1.07, 1.35), *p* = 0.0018], intravenous injection of vasopressors [aHR 1.54 (95% CI: 1.32, 1.79), *p* = 0.0001] and normal saline [aHR 1.18 (95% CI: 1.05, 1.33), *p* = 0.0066] were associated with a high risk of the outcome. In conclusion, this study showed that the use of pressor approaches during HD may be an independent risk factor for death.

## Introduction

Cardiovascular disease (CVD) is one of the leading causes of death in dialysis patients in Japan, and the CVD-caused death was 32.6%^1^. Because CVD is strongly associated with blood pressure, the control of blood pressure is one of the most essential strategies for hemodialysis (HD) patients.

It has been reported that post-HD systolic blood pressures higher than 180 mmHg and lower than 110 mmHg indicated a high risk of CVD-caused death in HD patients^2^. That is, the relationship between post-HD systolic blood pressure and CVD-caused death showed a U-shape. According to the Annual Dialysis Data Report 2005 of the Japanese Society for Dialysis Therapy (JSDT) Renal Data Registry (JRDR), which is a nationwide renal data registry and contains data of all dialysis patients (n = 232,226) in Japan, the percentages of HD patients with post-HD systolic blood pressures lower than 100 mmHg and higher than 180 mmHg were 5.0% and 5.3%, respectively^3^.

Hypotension is often encountered in HD patients^4^. HD-related hypotension includes as chronically sustained hypotension, intradialytic hypotension (IDH), and orthostatic hypotension^5^. The dialysis outcomes and practice patterns study (DOPPS) showed that the risk of death in HD patients is high at a pre-HD systolic blood pressure of lower than 130 mmHg^6^. HD-associated hypotension is a risk factor for death^7^. IDH is associated with CVD and CVD-caused death^4,8^.

When hypotension occurs during HD, HD continuation is sometimes difficult. Therefore, to prevent hypotension, its management is required such as discontinuation of ultrafiltration, enhancement of plasma refilling, administration of vasopressors, low-temperature dialysis, and changing to other methods of dialysis^5^. A systematic review of ten studies showed that post-HD systolic and diastolic blood pressures were increased by midodrine treatment^9^. In Japan, the most commonly used vasopressors per os (po) were midodrine hydrochloride, amezinium metilsulfate, etilefrine hydrochloride, and droxidopa. Etilefrine hydrochloride is also used as a vasopressor administered by intravenous injection (iv). Catecholamine preparations are used as vasopressors (iv), but at a low frequency. And a systematic review showed that low-temperature dialysis improves blood pressure during HD and reduces the rate of IDH^10^. However, a cohort study have reported that midodrine is associated with high risk of death^11^. The effects of the treatment of IDH may not be always beneficial.

Although pressor approaches are necessary for patients with hypotension, its effect on HD patients’ prognosis has not been clarified yet as far as our literature research has shown. Because a randomized controlled trial can hardly show which combination of pressor approaches is effective in preventing hypotension and improving HD patients’ prognosis, there is no evidence of how to use pressor approaches. Therefore, the aims of this study were to investigate the relationships between the use of pressor approaches and one-year all-cause death, and to determine the appropriate methods to use such approaches on the basis of JRDR data.

## Results

### Baseline characteristics

From the JRDR data, pressor approaches included low-temperature dialysis, the vasopressors (po), and the intravenous injection of medicines [normal saline, high-concentration sodium chloride solution, glycerin, vasopressors (iv)]. The subjects were categorized into the pressor and nonpressor groups on the basis of the use of pressor approaches. The baseline characteristics including biochemical data are shown in Table 1.

**Table 1:** Baseline characteristics.

	All	Pressor group	Nonpressor group	*p* value
N	29309	10775	18534	
Male (%)	17468 (59.6)	5568 (51.7)	11900 (64.2)	0.0001
Age (years)	64.5 ± 12.5	66.9 ± 11.9	63.1 ± 12.7	0.0001
CVD (%)	4976 (17)	2106 (19.5)	2870 (15.5)	0.0001
DM (%)	9226 (31.5)	4008 (37.2)	5218 (28.2)	0.0001
Depressor (%)	19495 (66.5)	6404 (59.4)	13091 (70.6)	0.0001
ACEI or ARB	12458 (42.5)	3911 (36.3)	8547 (46.1)	0.0001
CCB	14508 (49.5)	4511 (41.9)	9997 (53.9)	0.0001
Pressor (%)				
Low-temperature dialysis	2019 (6.9)	2019 (18.7)	0 (0)	
Normal saline	5125 (17.5)	5125 (47.6)	0 (0)	
Sodium chloride	2751 (9.4)	2751 (25.5)	0 (0)	
Glycerin	683 (2.3)	683 (6.3)	0 (0)	
Vasopressor (iv)	1662 (5.7)	1662 (15.4)	0 (0)	
Vasopressor (po)	5260 (17.9)	5260 (48.8)	0 (0)	
Vintage (years)	7.7 ± 6.3 5.8 (3, 10.3)	7.8 ± 6.4 5.8 (3, 10.2)	7.7 ± 6.2 5.8 (3.1, 10.5)	0.24
BMI (kg/m^2^)	21.0 ± 3.4	21.2 ± 3.6	20.9 ± 3.2	0.0001
Albumin (g/dL)	3.8 ± 0.4	3.7 ± 0.4	3.8 ± 0.4	0.0001
Creatinine (mg/dL)	10.8 ± 2.8	10.4 ± 2.7	11.1 ± 2.8	0.0001
CRP (mg/dL)	0.52 ± 1.68 0.12 (0.06, 0.39)	0.61 ± 1.99 0.17 (0.08, 0.5)	0.46 ± 1.46 0.1 (0.05, 0.3)	0.15
Hemoglobin level (g/dL)	10.3 ± 1.3	10.3 ± 1.4	10.3 ± 1.3	0.96
Fluid removal rate (%)	−4.4 ± 1.6	−4.5 ± 1.7	−4.3 ± 1.6	0.0001
Pre-HD SBP (mmHg)	154.9 ± 24.2	153.7 ± 25.9	155.6 ± 23.2	0.0001
Post-HD SBP (mmHg)	138.2 ± 24.4	133.2 ± 24.9	141.1 ± 23.6	0.0001
Minimum SBP (mmHg)	121.2 ± 22.5	112.5 ± 22.8	126.3 ± 20.7	0.0001
Pre-HD DBP (mmHg)	80.5 ± 13.6	79.1 ± 14	81.3 ± 13.3	0.0001
Post-HD DBP (mmHg)	74.9 ± 13.5	72.2 ± 13.7	76.4 ± 13.1	0.0001
Minimum DBP (mmHg)	66 ± 13.5	61.9 ± 13.9	68.4 ± 12.7	0.0001
All-cause death (%)	1894 (6.5)	963 (8.9)	931 (5)	0.0001
CVD-caused death (%)	852 (2.9)	444 (4.1)	408 (2.2)	0.0001
Infection-caused death (%)	177 (0.6)	96 (0.9)	81 (0.4)	0.0001

Variables are expressed as mean ± standard deviation. Vintage and CRP are also shown as median and interquartile range. Intergroup comparisons of parameters were performed using the chi-square test, t-test, and Mann-Whitney U test as appropriate.

Abbreviations: CVD, cardiovascular disease; DM, diabetes mellitus as a cause of end-stage renal disease; ACEI, angiotensin-converting-enzyme inhibitor; ARB, angiotensin II receptor blocker; CCB, calcium channel blocker; BMI, body mass index; CRP, C-reactive protein; HD, hemodialysis; SBP, systolic blood pressure; DBP, diastolic blood pressure.

The pressor group showed larger numbers of females, subjects with CVD, and subjects with diabetes mellitus (DM) as the cause of end-stage renal disease (ESRD); older age; higher body mass index (BMI); lower serum albumin and creatinine levels; higher serum C-reactive protein (CRP) level; and higher fluid removal rate. The mean pre-HD, post-HD blood pressures and minimum systolic and diastolic blood pressures during HD were lower in the pressor group than in the nonpressor group.

The multivariate logistic regression model showed that the pressor group tended to include a high rate of females, older age, CVD, DM, long vintage, high BMI, low serum albumin and creatinine levels, high serum CRP level, and high hemoglobin level, and high fluid removal rate, and low blood pressures (Table 2).

**Table 2:** Pressor approaches and related factors.

	Odds ratio (95% CI)	*p* value
Model 1		
Male (%)	0.56 (0.53, 0.59)	0.0001
Age (years)	1.02 (1.02, 1.03)	0.0001
CVD (%)	1.17 (1.09, 1.25)	0.0001
DM (%)	1.54 (1.45, 1.64)	0.0001
Ln(vintage)	1.09 (1.06, 1.13)	0.0001
BMI (kg/m^2^)	1.06 (1.05, 1.07)	0.0001
Albumin (g/dL)	0.92 (0.86, 0.99)	0.021
Creatinine (mg/dL)	0.98 (0.97, 0.99)	0.011
Ln(CRP)	1.16 (1.14, 1.19)	0.0001
Hemoglobin level (g/dL)	1.08 (1.05, 1.10)	0.0001
Fluid removal rate (%)	0.89 (0.87, 0.90)	0.0001
pre-HD SBP	0.995 (0.993, 0.996)	0.0001
Model 2 (post-HD SBP)	0.986 (0.985, 0.987)	0.0001
Model 3 (minimum SBP)	0.972 (0.971, 0.973)	0.0001
Model 4 (pre-HD DBP)	0.996 (0.994, 0.998)	0.0001
Model 5 (post-HD DBP)	0.984 (0.982, 0.986)	0.0001
Model 6 (minimum DBP)	0.969 (0.967, 0.971)	0.0001

Factors related to the use of pressor approaches were evaluated by multivariate logistic regression models (Models 1 to 6) adjusted for baseline characteristics such as gender, age, CVD, DM, ln(vintage), BMI, serum albumin, and creatinine levels, ln(CRP), hemoglobin level, fluid removal rate, and blood pressures. The values are expressed as odds ratios (95% CIs) and *p* values.

Abbreviations: CI, confidence interval; CVD, cardiovascular disease; DM, diabetes mellitus as a cause of end-stage renal disease; BMI, body mass index; CRP, C reactive protein; HD, hemodialysis; SBP, systolic blood pressure; DBP, diastolic blood pressure.

### Risk of death and use of pressor approaches

**Figure 1:**
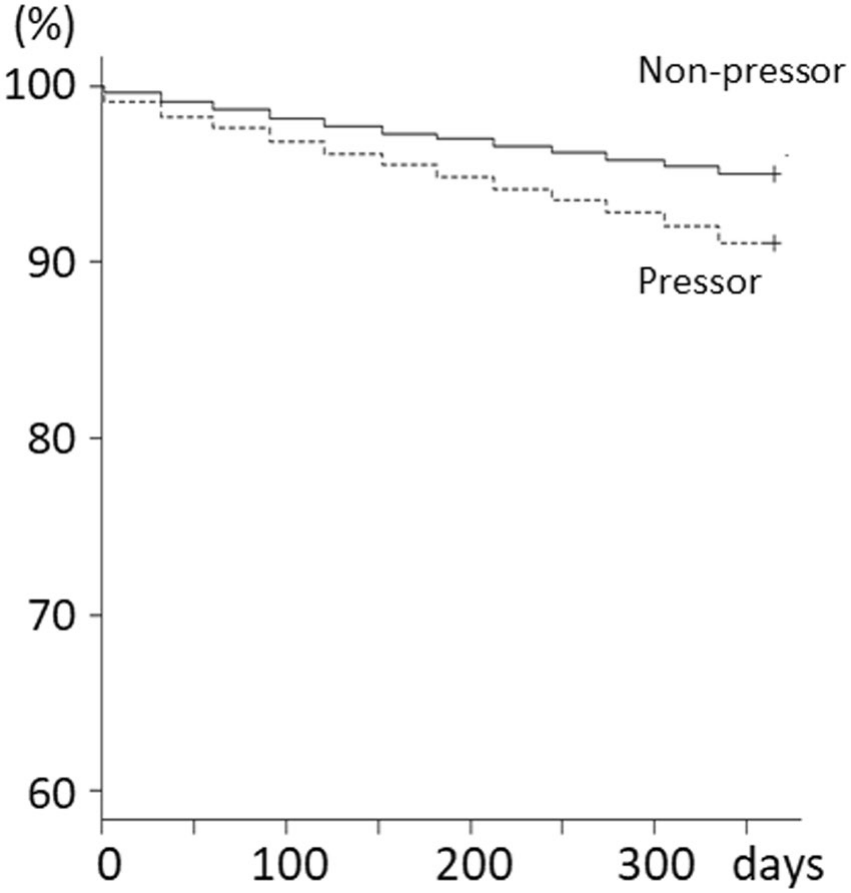
Association between use of pressor approaches and risk of all-cause death. The Kaplan-Meier survival curve showed a lower survival probability in the pressor group than in the nonpressor group (Log-rank and Wilcoxon tests, *p* = 0.0001). Solid line is the nonpressor group. Dashed line is the pressor group. Abbreviations: pressor, pressor group; nonpressor, nonpressor group.

All-cause, CVD-caused, and infection-caused deaths were more frequently observed in the pressor group than in the nonpressor group (Table 1). U-shaped relationships were observed between blood pressure and the risk of all-cause death (Supplementary Fig. S1). Kaplan-Meier analysis showed that the pressor group had a higher mortality rate than the nonpressor groups (Fig. 1). Cox proportional hazards models (PHMs) and adjusted Cox PHMs showed that the pressor group showed a high risk of all-cause death (Table 3). Competing risk regression models showed that the risks of CVD- and infection-caused deaths in the pressor group were higher than those in the nonpressor group (Table 4).

**Table 3:** Pressor approaches and risk of all-cause death.

Models	Hazard ratio (95%CI) *p* value
Crude	1.81 (1.66, 1.98) *p* = 0.0001
Adjusted models	
Model 1 Baseline and pre-HD SBP	1.33 (1.21, 1.47) *p* = 0.0001
Model 2 Baseline and post-HD SBP	1.34 (1.21, 1.47) *p* = 0.0001
Model 3 Baseline and minimum SBP	1.29 (1.17, 1.42) *p* = 0.0001
Model 4 Baseline and pre-HD DBP	1.34 (1.22, 1.47) *p* = 0.0001
Model 5 Baseline and post-HD DBP	1.34 (1.22, 1.47) *p* = 0.0001
Model 6 Baseline and minimum DBP	1.30 (1.18, 1.43) p = 0.0001

HRs (95% CIs) are given by Cox proportional hazards models adjusted for baseline characteristics such as gender, age, CVD, DM, ln(vintage), BMI, serum albumin, and creatinine levels, ln(CRP), hemoglobin level, and fluid removal rate, and blood pressures.

Abbreviations: HR, hazard ratio; CI, confidence interval; HD, hemodialysis; SBP, systolic blood pressure; DBP, diastolic blood pressure; baseline and pre-HD SBP, a Cox proportional hazards model adjusted for baseline characteristics and pre-HD SBP.

**Table 4:** Pressor approaches and risks of CVD- and infection-caused deaths.

Model	Hazard ratio (95%CI) *p* value
CVD-caused death	
Crude	1.89 (1.66, 1.98) *p* = 0.0001
Adjusted models	
Model 1 Baseline and pre-HD SBP	1.42 (1.23, 1.64) *p* = 0.0001
Model 2 Baseline and post-HD SBP	1.45 (1.25, 1.67) *p* = 0.0001
Model 3 Baseline and minimum SBP	1.41 (1.22, 1.64) *p* = 0.0001
Model 4 Baseline and pre-HD DBP	1.42 (1.23, 1.64) *p* = 0.0001
Model 5 Baseline and post-HD DBP	1.44 (1.25, 1.66) *p* = 0.0001
Model 6 Baseline and minimum DBP	1.41 (1.22, 1.63) p = 0.0001
Infection-caused death	
Crude	1.89 (1.66, 1.98) *p* = 0.0001
Adjusted models	
Model 1 Baseline and pre-HD SBP	1.43 (1.05, 1.95) *p* = 0.022
Model 2 Baseline and post-HD SBP	1.43 (1.04, 1.96) *p* = 0.027
Model 3 Baseline and minimum SBP	1.26 (0.92, 1.73) *p* = 0.16
Model 4 Baseline and pre-HD DBP	1.44 (1.06, 1.97) *p* = 0.020
Model 5 Baseline and post-HD DBP	1.39 (1.02, 1.90) *p* = 0.036
Model 6 Baseline and minimum DBP	1.34 (0.98, 1.83) p = 0.069

Values are given as HRs (95% CIs). Competing risk regression models (Models 1 to 6) were adjusted for baseline characteristics such as gender, age, CVD, DM, ln(vintage), BMI, serum albumin, and creatinine levels, ln(CRP), hemoglobin level, fluid removal rate, and blood pressures.

Abbreviations: HR, hazard ratio; CI, confidence interval; HD, hemodialysis; SBP, systolic blood pressure; DBP, diastolic blood pressure.

### Propensity score-matched analysis and Bayesian network (BN)

There were no significant differences between the baseline characteristics of the matched pressor and matched nonpressor groups except for post-HD and minimum systolic and diastolic blood pressures (Supplementary Table 1). Kaplan-Meier analysis showed that the matched pressor group had a higher mortality rate than the matched nonpressor groups (Supplementary Fig. S2). Cox PHMs showed that the use of pressor approaches was independently associated with a higher risk of the all-cause death (Supplementary Table 2).

**Figure 2:**
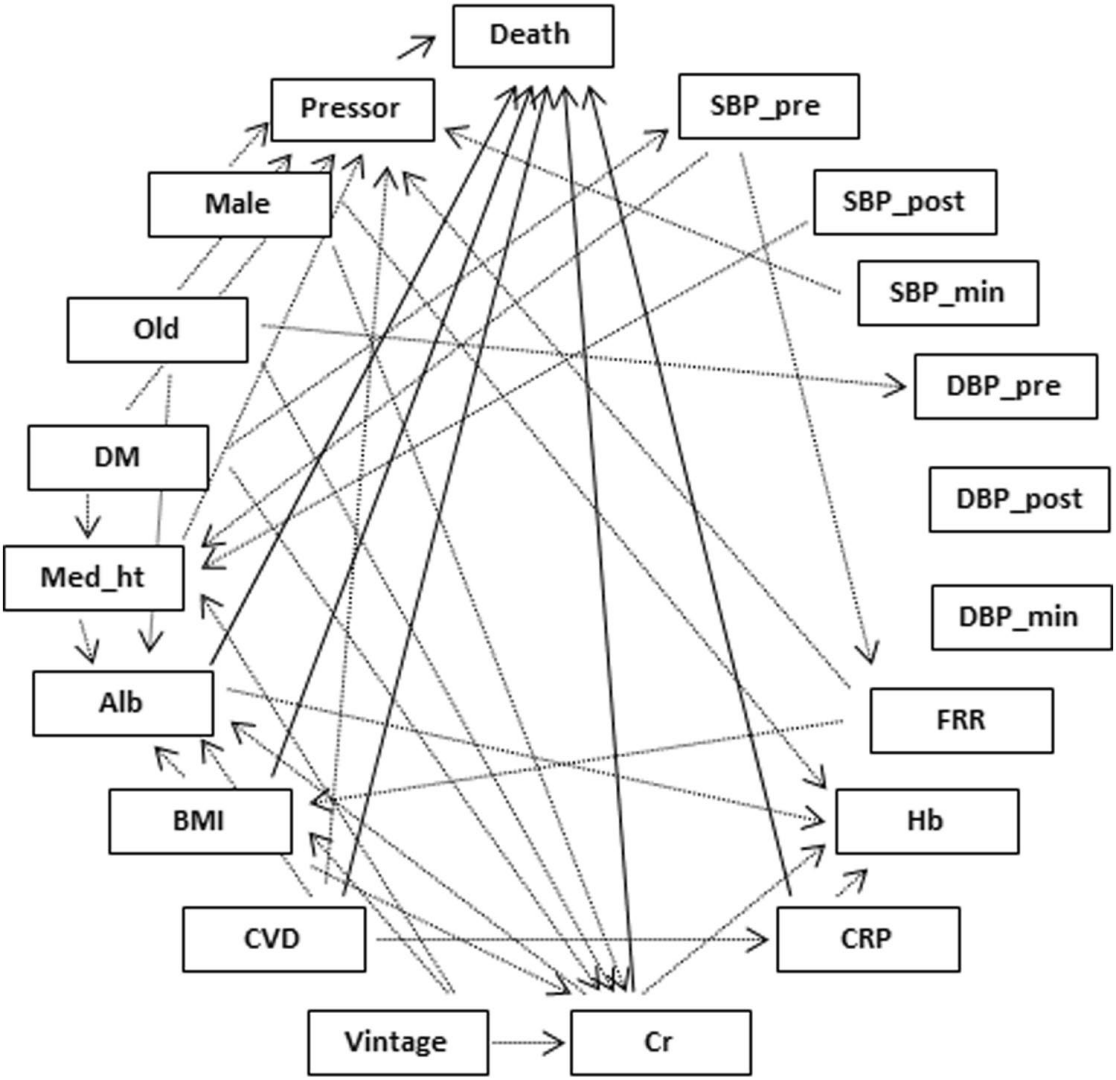
Causal Bayesian network structure. The directed acyclic graph shows the causal relationships between variables where nodes and links represent variables and causal relationships, respectively. Solid arrows indicate factors to death. And dotted lines show other relationships. Abbreviations: Death, all-cause death; Pressor, pressor approaches; Old, age more than 65 years; DM, diabetes mellitus as a cause of end-stage renal disease; Med_ht, depressor; Alb, serum albumin level; BMI, body mass index; CVD, cardiovascular disease; Cr, serum creatinine level; CRP, C-reactive protein; Hb, hemoglobin; FRR, fluid removal rate; SBP, systolic blood pressure; DBP, diastolic blood pressure; Pre, pre-hemodialysis; Post, post-hemodialysis; min, minimum.

Using the dataset of all subjects, BN including all blood pressures showed causal relationships between variables (Fig. 2). All-cause death was affected by the use of pressor approaches; serum albumin, creatinine, and CRP levels; BMI; and CVD. The use of pressor approaches were affected by the minimum systolic blood pressure, being male, older age, DM, use of depressors, CVD, and fluid removal rate. Each BN including each blood pressure showed similar relationships between blood pressure, pressor approaches, and all-cause death (results not shown).

BN also showed the factors associated with the use of pressor approaches such as gender, old age, a history of CVD, DM, high fluid removal rate, and low blood pressure.

### Types of pressor approaches and risk of all-cause death

There were weak differences in the baseline characteristics between the uses of pressor approaches (Table 5). The groups of glycerin, vasopressors (iv) and (po) showed older age. The groups of normal saline and high-concentration sodium chloride showed smaller numbers of subjects with CVD. The glycerin group showed smaller numbers of subjects with DM and lower fluid removal rate. The vasopressor (iv) group showed lower blood pressures. And the groups of glycerin and vasopressors (iv) showed higher risk of all-cause and CVD-caused death.

**Table 5:** Difference in baseline characteristics between pressor approaches.

	Low-temperature dialysis	Normal saline	Sodium chloride	Glycerin	Vasopressor (iv)	Vasopressor (po)
N	2019	5125	2751	683	1662	5260
Male (%)	1065 (52.7)	2673 (52.2)	1376 (50)	330 (48.3)	809 (48.7)	2527 (48)
Age (years)	66.8 ± 12.2	66.4 ± 12.2	67.1 ± 11.8	67.9 ± 12.2	68.5 ± 11.7	68.2 ± 11.3
CVD (%)	454 (22.5)	987 (19.3)	528 (19.2)	160 (23.4)	364 (21.9)	1073 (20.4)
DM (%)	765 (37.9)	1843 (36)	1065 (38.7)	217 (31.8)	668 (40.2)	2174 (41.3)
Depressor (%)	1222 (60.5)	3170 (61.9)	1637 (59.5)	424 (62.1)	907 (54.6)	2871 (54.6)
Vintage (years)	7.2 ± 5.7 5.7 (3, 9.5)	7.9 ± 6.7 5.7 (3, 10.5)	8 ± 6.7 5.9 (3.1, 10.8)	7.9 ± 6.6 6.1 (2.9, 11)	7.4 ± 6.3 5.4 (2.9, 9.6)	7.4 ± 6.2 5.5 (3, 9.6)
BMI (kg/m^2^)	21.1 ± 3.5	21 ± 3.5	21.2 ± 3.7	21.1 ± 3.8	21 ± 3.7	21.5 ± 3.7
Albumin (g/dL)	3.7 ± 0.4	3.8 ± 0.4	3.8 ± 0.4	3.7 ± 0.5	3.7 ± 0.5	3.7 ± 0.4
Creatinine (mg/dL)	10.4 ± 2.8	10.5 ± 2.8	10.3 ± 2.7	10.1 ± 2.7	10 ± 2.7	10.1 ± 2.6
CRP (mg/dL)	0.7 ± 2.8 0.19 (0.09, 0.5)	0.6 ± 2.01 0.16 (0.08, 0.46)	0.6 ± 1.52 0.19 (0.09, 0.5)	0.82 ± 3.53 0.23 (0.1, 0.69)	0.81 ± 2.77 0.2 (0.1, 0.6)	0.64 ± 2.1 0.2 (0.09, 0.51)
Hemoglobin level (g/dL)	10.2 ± 1.6	10.2 ± 1.4	10.3 ± 1.3	10.3 ± 1.4	10.2 ± 1.5	10.4 ± 1.4
Fluid removal rate (%)	−4.5 ± 1.6	−4.5 ± 1.7	−4.6 ± 1.8	−4.3 ± 1.6	−4.5 ± 1.6	−4.5 ± 1.6
Pre-HD SBP (mmHg)	155.7 ± 25.8	154.1 ± 25.5	153 ± 26.7	150.5 ± 27.2	150 ± 27.2	153.7 ± 26.8
Post-HD SBP (mmHg)	135.2 ± 24.7	132.8 ± 25.1	132.1 ± 25.4	132 ± 26.6	129.6 ± 25.4	133.1 ± 25.3
Minimum SBP (mmHg)	113.7 ± 22.4	109.6 ± 23.6	109.3 ± 23.4	109.1 ± 24.1	106.9 ± 23.2	112.6 ± 22.9
Pre-HD DBP (mmHg)	79.7 ± 14.2	78.8 ± 14	78.5 ± 14.4	77.7 ± 14.4	77.3 ± 14.3	79.1 ± 14.4
Post-HD DBP (mmHg)	72.7 ± 13.6	71.8 ± 13.8	71.5 ± 13.7	71 ± 14.6	69.8 ± 13.8	72.2 ± 14
Minimum DBP (mmHg)	62.3 ± 14	60.5 ± 14.4	60.5 ± 13.8	59.5 ± 14.3	58.8 ± 14.9	61.7 ± 14.1
All-cause death (%)	193 (9.6)	456 (8.9)	263 (9.6)	91 (13.3)	240 (14.4)	514 (9.8)
CVD-caused death (%)	92 (4.6)	202 (3.9)	112 (4.1)	44 (6.4)	114 (6.9)	237 (4.5)
Infection-caused death (%)	23 (1.1)	46 (0.9)	27 (1)	7 (1)	22 (1.3)	52 (1)

Variables are expressed as mean ± standard deviation. Vintage and CRP are also shown as median and interquartile range. Because the subjects in the groups of the use of pressor approaches are repeated, statistical difference was not evaluated between the groups.

Abbreviations: CVD, cardiovascular disease; DM, diabetes mellitus as a cause of end-stage renal disease; BMI, body mass index; CRP, C-reactive protein; HD, hemodialysis; SBP, systolic blood pressure; DBP, diastolic blood pressure.

The relationship between the risk of all-cause death and the type of pressor approaches was evaluated by multivariate Cox PHMs using the dataset of all subjects (Table 6). The use of normal saline, vasopressor (iv), and vasopressor (po) was associated with a high risk of all-cause death.

**Table 6:** Type of pressor approaches and risk of all-cause death.

	Crude HR (95%CI) *p* value	Adjusted HR (95%CI) *p* value
Low-temperature dialysis	1.14 (0.97, 1.34) *p* = 0.12	1.01 (0.86, 1.19) *p* = 0.87
Normal saline	1.23 (1.09, 1.38) *p* = 0.0005	1.18 (1.05, 1.33) *p* = 0.0066
Sodium chloride	1.10 (0.95, 1.27) *p* = 0.20	1.03 (0.88, 1.19) *p* = 0.74
Glycerin	1.21 (0.95, 1.53) *p* = 0.12	1.05 (0.83, 1.33) *p* = 0.69
Vasopressor (iv)	1.91 (1.64, 2.22) *p* = 0.0001	1.54 (1.32, 1.79) *p* = 0.0001
Vasopressor (po)	1.43 (1.28, 1.59) *p* = 0.0001	1.20 (1.07, 1.35) *p* = 0.0018

Values are given as HRs (95% CI). The Cox proportional hazards model was adjusted for baseline characteristics such as gender, age, CVD, DM, ln(vintage), BMI, serum albumin, and creatinine levels, ln(CRP), hemoglobin level, fluid removal rate, and pre-HD systolic blood pressure.

Abbreviations: HR, hazard ratio; CI, confidence interval.

## Discussion

This study using large-scale cohort data, showed that the pressor approaches were independent risk factors for all-cause death. Because observational analyses of pressor approaches and outcomes are subject to bias owing to unmeasured confounders, propensity score matched analysis was used in this study to minimize the bias, which showed that the pressor group had high risk of all-cause death. BN suggested that all-cause death was directly affected by the use of pressor approaches. From these results, it was suggested that the use of pressor approaches was an independent risk factor for death. There has been no report on the harmful effects of pressor approaches on patients’ prognosis as far as we searched the literature of prospective interventional studies.

IDH is caused by many factors, such as low dry weight, excessive ultrafiltration, decrease in osmolality, and autonomic neuropathy^5,12,13^. Pressor approaches are usually administered to prevent and control IDH^5,13^. In this study, the harmful effects of administration of normal saline, vasopressors (iv), and vasopressors (po) on patients’ prognosis were observed. On the other hand, low-temperature dialysis, and injection of high-concentration sodium chloride solution and high-concentration glycerin were not associated with the risk of all-cause death. Normal saline is usually given to replace intravascular volume as a method of acute management of IDH, and to effectively maintain blood pressure^14^. The harmful effects of sodium loading have been reported^15^. Some of the harmful effects of normal saline are the acute change in circulating plasma volume, poor cardiac function, and complications of IDH. However, considering that the use of high-concentration sodium chloride solution and high-concentration glycerin was not associated with the risk of all-cause death, intravascular volume loading may have a strong effect on the risk of all-cause death, and the effect of sodium loading may not be strong.

Our study showed the relationship between the use of vasopressors and the risk of all-cause death. A cohort study showed that the use of midodrine is associated with risk of death^11^. There were also reports that midodrine hydrochloride and amezinium metilsulfate might have worsened the leg ulcers in HD patients^16,17^. IDH causes hypoperfusion in organs causing myocardial infarction, stroke, and bowel ischemia, and leads to myocardial fibrosis and cardiac remodeling^18^. HD-induced myocardial stunning is associated with the development of heart failure and increased risk of all-cause death in HD patients^19–21^. IDH is associated with low cardiac index and high peripheral resistance^22^. Considering the mechanism of action of vasopressors, vasoconstriction induced by the vasopressors may decrease the blood supply to peripheral arteries, and worsen HD patients’ prognosis. If an HD patient’s blood pressure can be maintained during HD by pressor approaches excluding the use of vasopressors, it would be better to avoid using vasopressors as much as possible.

Low-temperature dialysis is used for IDH by increasing peripheral vascular resistance and improving cardiac output. In our study, low-temperature dialysis was not associated with a risk of all-cause death. A systematic review of 26 studies showed that low-temperature dialysis reduces the rate of IHD and increases intradialytic mean arterial pressure^10^. The candidate mechanisms underlying the favorable effects of low-temperature dialysis on HD are the improvement of intradialytic hemodynamics by the preservation of cardiac output and central blood volume, and the slowing of the progression of HD-associated cardiomyopathy, brain protection, and improvement of nocturnal sleep^23–27^. These effects are different from those of vasopressors, and may affect patients’ prognosis. Although discomfort is more often observed in low-temperature dialysis than in normal-temperature dialysis, low-temperature dialysis can be a candidate therapy to prevent IDH. Low-temperature dialysis is also recommended by the guidelines^5,13^.

In this study, logistic regression models and BN were used to investigate the factors associated with the use of pressor approaches. Logistic regression models and BN showed the common factors such as gender, old age, a history of CVD, DM, high fluid removal rate, and low blood pressure. These characteristics were in accordance with previous studies^28,29^. Patients with severe hypotension that is difficult to control have a high risk of all-cause death. According to previous studies, comorbid conditions such as coronary artery disease, systolic dysfunction, left ventricular hypertrophy, and autonomic dysfunction are observed in patients with IDH^18,28–31^. These factors indicate the difficulty of fluid removal, and hidden risks of cardiac dysfunction in such patients.

No relationship between serum albumin level and the use of pressor approaches was observed. However, this does not suggest that a low serum albumin level is not an important risk factor, but means that hypotension due to hypoalbuminemia may be difficult to control using pressor approaches. Albumin preparations are blood products and are difficult to use for daily HD. Hypoalbuminemia is caused by not only malnutrition but also inflammation and comorbid conditions^32^. To control hypoalbuminemia, it is important to identify its underlying causes and treat them.

In this study, the numbers of the subjects using vasopressor (po) and normal saline were higher than those using other pressor approaches. This finding might reflect a tendency that in Japan, a vasopressor (po) is commonly used for IDH as the first choice, and that normal saline is used when a patient’s blood pressure suddenly decreases. However, considering these results of our study and the JSDT and K/DOQI guidelines, the acute management of IDH by increasing intravascular volume using normal saline and use of vasopressors should be avoided^5,11,13^. Moderate fluid removal should be a key therapeutic strategy, for which reassessment of dry weight, regulation of ultrafiltration rate, extended hours of HD, and changes to other modes such as extracorporeal ultrafiltration method, hemodiafiltration, daily HD, and nocturnal HD may be effective^33,34^.

This study has several limitations. First, because of the observational nature of this study, the results may be biased by unmeasured confounders. To overcome this limitation, propensity matching analysis was conducted. However, JRDR data did not include the baseline characteristics of HD patients at the time of their first use of pressor approaches. Thus, to examine the causal relationship between vasopressors and the risk of all-cause death, BN was conducted, and these analyses showed similar results. Although propensity matching analysis and BN were conducted, there remains some bias. For example, pressor approaches were often used for patients with high risk of death. Second, we did not include the patients with missing data in this study, which might have caused selection bias. Third, the JRDR data did not standardize the methods of blood pressure measurement. However, the methods were almost similar throughout Japan. Therefore, despite the difference in the method of measuring blood pressure, the relationship between pressor approaches and the risk of all-cause death is considered robust. Fourth, in this study, the use of high-concentration sodium chloride and normal saline, which increase intravascular volume, tended to be avoided in patients with CVD. There were biases in the indication of the use of pressor approaches in this study, and biases in the difference in sample size between pressor approaches. However, although the glycerin group showed a higher risk of death than other groups, the Cox proportional hazard models showed no statistically significant relationship between the use of glycerin and the risk of death. The indication bias might not be strong and might be adjusted in the models. Because the details on the use of pressor approaches were not included in the JRDR data, we were unable to evaluate the effects of the patterns of the use of pressor approaches. Randomized controlled trials are needed to evaluate the effects of the use of pressor approaches on dialysis patients’ mortality. Fifth, there was some bias due to the medical situations with regard to the use of pressor approaches in Japan such as their coverage by national health insurance and health care policies at facilities. Because there may be differences in IDH treatment between countries, to show external validity, comparison of the use of pressor approaches between countries is needed. Sixth, although IDH is pathophysiologically associated with cardiac output, arterior vasoconstrictrion, and autonomic system, in this study, we lacked pathophysiological data or details on CVD death such as myocardial infarction and arrythmia^4^. More studies are needed to investigate the pathophysiological mechanism of IDH and medication. Seventh, there were some differences in the factors associated with the use of pressor approaches between the logistic regression models and BN such as increases in BMI and hemoglobin level. The logistic regression models might not be appreciable to the complex relationships between variables. More details on medication are needed to determine the mechanisms of development of IDH.

In conclusion, this study showed that the HD patients for whom pressor approaches were used during HD have high risk of death. In particular, the use of normal saline, and vasopressors is associated with a poor prognosis. To improve HD patients’ prognosis, it may be effective to prevent a situation that requires acute management of IDH.

## Methods

### Dataset

This is a prospective cohort study of maintenance HD patients using JRDR data. JSDT has been conducting annual year-end surveys of dialysis facilities in Japan since 1968. The JRDR data from 2005 to 2006 were used in this study. This study was approved by the ethics committee of JSDT and was exempt from the need to obtain informed consent from participants (JSDT No. 18). The study was performed in accordance with the relevant guidelines and the Declaration of Helsinki.

The subjects of this cohort study were the 245,441 patients. The exclusion criteria were as follows: patients younger than twenty years; patients on hemodiafiltration, hemofiltration, or peritoneal dialysis; patients with missing values or outlier values of laboratory data; patients who had a limb amputated; and patients with a HD vintage of less than one year. Thus, 29,309 subjects were included in the analysis. The sample size was evaluated to maximize statistical power.

The baseline data were as follows: gender; age; history of CVD; DM as a cause of ESRD; vintage; post-HD BMI; serum albumin, creatinine, and CRP levels; hemoglobin level; and fluid removal rate. The laboratory data were measured before HD. Fluid removal rate (%) was calculated as follows: (post-HD weight − pre-HD weight)/pre-HD weight × 100. Data on Pre-HD, post-HD blood pressures and minimum systolic and diastolic blood pressures during HD were collected. In this study, methods of blood pressure measurement were not standardized, because JDRD data were collected by survey of dialysis facilities. However, the general method of blood pressure measurement in Japan at that time was as follows^5^. Blood pressure was measured under fixed conditions in either the seated or supine position depending on the setup at each facility. Pre-HD blood pressure was measured before the start of dialysis. Post-HD blood pressure was measured just before returning the blood at the end of dialysis. The lowest blood pressure was recorded as the minimum blood pressure during HD. Information on depressors [angiotensin-converting-enzyme inhibitors (ACEIs) and angiotensin II receptor blockers (ARBs)] used was collected. Information on methods used for raising blood pressure (pressors) was also collected, namely, low-temperature dialysis, vasopressors (po), and medicines for intravenous injection (normal saline, high-concentration sodium chloride solution, glycerin, vasopressors iv).

The primary outcome was all-cause death within one year. CVD- and infection-caused deaths within one year were also evaluated. Subjects were categorized on the basis of the use of pressor approaches: the pressor and nonpressor groups.

### Statistical analyses

Normally distributed variables are presented as mean ± standard deviation; otherwise, the median and interquartile ranges are presented. Highly skewed variables were transformed with the natural logarithm function prior to their use in models [ln(vintage), ln(CRP)]. Intergroup comparisons of parameters were performed using the chi-square test, t-test, and Mann-Whitney U test as appropriate after the F-test of equality of variances between the groups.

Using the Kaplan–Meier method, we compared the pressor and nonpressor groups in terms of the incidence of the primary outcome, and statistical significance was evaluated using the log-rank and Wilcoxon tests. A multivariate Cox PHM was used to evaluate the relationships of the pressor and nonpressor groups with mortality. This multivariate Cox PHM was adjusted for baseline characteristics [gender, age, CVD, DM, ln(vintage), BMI, serum albumin, and creatinine levels, ln(CRP), hemoglobin level, and fluid removal rate, and use of depressors] and blood pressures using the splines of the blood pressures. The results were presented as HRs with 95% confidence intervals (CIs). In the analysis of competing risks of cause-specific death, Fine and Gray competing risk regression models adjusted for the baseline characteristics were also examined.

A propensity score-matched cohort for the use of pressor approaches was conducted. Possible confounders were chosen for baseline characteristics, including baseline characteristics [gender, age, CVD, DM, ln(vintage), BMI, serum albumin and creatinine levels, ln(CRP), hemoglobin level, fluid removal rate, and use of depressors], and pre-HD systolic blood pressure. The predicted probability of the use of pressor approaches was calculated by applying a logistic regression model including these possible confounders. Next, we performed propensity score matching using the following algorithm: 1:1 nearest neighbor match within a caliper width defined as ±0.2 of a standard deviation (SD) of the logit of the propensity score and no replacement. Comparisons of baseline characteristics were based on the chi-square test, t-test, Mann-Whitney U, and standardized differences. The incidence of the primary outcome was compared between the matched pressor and nonpressor groups using the Kaplan–Meier method. A Cox PHM was used to evaluate the association between the matched pressor and nonpressor groups and the primary outcome. Adjustment in the multivariate Cox PHM was demonstrated for variables that showed absolute standardized differences (10% in baseline characteristics) between the matched pressor and nonpressor groups. The relationship between the types of pressor approaches and the risk of all-cause death was also evaluated using multivariate Cox PHMs adjusted for baseline characteristics and blood pressures.

BN is a kind of probabilistic graphical model that shows variables and their causal relationships via a directed acyclic graph, and represents the probabilistic relationships between diseases and symptoms. BN was used to evaluate the relationship between the variables described above. The incremental association Markov blanket method was used for structure learning algorithm for BN. The resulting directed acyclic graph was interpreted as the causal BN. Continuous variables were discretized by cutoff levels determined by receiver operating characteristic curves for the prediction of the primary outcome. Blood pressures were categorized as follows: systolic blood pressures, 100 mmHg, 150 mmHg, and 200 mmHg; diastolic blood pressures, 50 mmHg, 100 mmHg. These analyses were conducted using SAS version 9.4 (SAS, Inc., NC, USA) and R version 3.4.1 (R project for Statistical Computing, Vienna, Austria). Statistical significance was defined as *p* < 0.05.

## Supplementary information

Supplementary information

## Data Availability

The datasets generated during and/or analyzed during the current study cannot be publicly available because they are owned by JSDT. Please ask JSDT about data availability. The summary of the data has been opened on the JSDT home page (http://www.jsdt.or.jp/english/).
