# Supplementary material for: Use of vasopressor for dialysis-related hypotension is a risk factor for death in hemodialysis patients: Nationwide cohort study
Source: Sci Rep. 2019 Mar 4;9:3362. doi: 10.1038/s41598-019-39908-6 (PMC6399330; doi:10.1038/s41598-019-39908-6)
Supplement: Supplementary file 1 — Supplementary information [file 41598_2019_39908_MOESM1_ESM.docx]

**Use of vasopressor for dialysis-related hypotension is a risk factor for death in hemodialysis patients: Nationwide cohort study**

Eiichiro Kanda^1^, Yuki Tsuruta^2^, Kan Kikuchi^3^, Ikuto Masakane^4^

1. Medical Science, Kawasaki Medical School, Okayama, Japan.

2. Tsuruta Itabashi Clinic, Tokyo, Japan.

3. Shimoochiai Clinic, Tokyo, Japan.

4. Department of Nephrology, Honcho Yabuki Clinic, Yamagata, Japan.

Supplementary Table 1. Baseline characteristics according to use of pressor approaches in propensity-matched subjects. Page 3.

Supplementary Table 2. Pressor approaches and risks of all-cause death in the matched dataset. Page 5.

Supplementary Figure S1. Blood pressure and risk of all-cause death. Page 6.

Supplementary Figure S2. Association between use of pressor approaches and risk of all-cause death in matched subjects. Page 7.

|  | All | Matched pressor group | Matched nonpressor group | *p* value | *d* (%) |
| --- | --- | --- | --- | --- | --- |
| N | 17840 | 8920 | 8920 |  |  |
| Male (%) | 9875(55.4) | 4920(55.2) | 4955(55.5) | 0.61 | 0.79 |
| Age (years) | 66.4±11.9 | 66.4±11.9 | 66.4±11.7 | 0.91 | 0.17 |
| CVD (%) | 3364(18.9) | 1685(18.9) | 1679(18.8) | 0.92 | 0.17 |
| DM (%) | 6223(34.9) | 3118(35) | 3105(34.8) | 0.85 | 0.31 |
| Depressor (%) | 11033(61.8) | 5487(61.5) | 5546(62.2) | 0.37 | 1.36 |
| Vintage (years) | 7.7±6.3 5.8(3.5,10.3) | 7.8±6.5 5.8(3.0,10.3) | 7.6±6.2 5.7(3.1,10.3) | 0.46 | 3.01 |
| BMI (kg/m^2^) | 21.1±3.4 | 21.1±3.5 | 21.1±3.4 | 0.70 | 0.57 |
| Albumin (g/dL) | 3.8±0.4 | 3.8±0.4 | 3.8±0.4 | 0.94 | 0.11 |
| Creatinine (mg/dL) | 10.5±2.7 | 10.5±2.7 | 10.5±2.7 | 0.67 | 0.63 |
| CRP (mg/dL) | 0.61±1.70 0.18(0.1,0.5) | 0.58±1.64 0.19(0.1,0.5) | 0.64±1.76 0.17(0.09, 0.5) | 0.12 | 3.35 |
| Hemoglobin level (g/dL) | 10.3±1.3 | 10.3±1.4 | 10.3±1.3 | 0.18 | 2.02 |
| Fluid removal rate (%) | -4.5±1.6 | -4.5±1.6 | -4.5±1.6 | 0.54 | 0.91 |
| Pre-HD SBP | 154.1±24.7 | 154.0±25.6 | 154.2±23.7 | 0.57 | 0.85 |
| Post-HD SBP | 136.4±24.6 | 133.4±24.8 | 139.4±24.0 | 0.0001 | 24.4 |
| Minimum SBP | 118.6±22.5 | 112.9±22.7 | 124.2±20.9 | 0.0001 | 51.5 |
| Pre-HD DBP | 79.5±13.5 | 79.6±14.0 | 79.4±13.1 | 0.25 | 1.71 |
| Post-HD DBP | 73.5±13.4 | 72.6±13.7 | 74.5±13.0 | 0.0001 | 14.0 |
| Minimum DBP | 66.5±13.3 | 62.4±13.7 | 66.6±12.4 | 0.0001 | 32.3 |
| All-cause death (%) | 1368(7.7) | 769(8.6) | 599(6.7) | 0.0001 | 7.17 |
| CVD-caused death (%) | 616(3.5) | 358(4) | 258(2.9) | 0.0001 | 6.14 |
| Infection-caused death (%) | 134(0.8) | 75(0.8) | 59(0.7) | 0.19 | 2.08 |

**Supplementary Table 1. Baseline characteristics according to use of pressor approaches in propensity-matched subjects**

Variables are expressed as mean±standard deviation. Vintage and CRP are also shown as median and interquartile range. Intergroup comparisons of parameters were performed using the chi-square test, t-test, and Mann-Whitney U test as appropriate.

Abbreviations: *d*, standardized difference; CVD, cardiovascular disease; DM, diabetes mellitus as a cause of end-stage renal disease; BMI, body mass index; CRP, C-reactive protein; HD, hemodialysis; SBP, systolic blood pressure; DBP, diastolic blood pressure.

| Model | Hazard ratio (95%CI) *p* value |
| --- | --- |
| Crude | 1.29 (1.16, 1.44) *p*=0.0001 |
| Adjusted models |  |
| Model 1 Post-HD SBP | 1.30 (1.17, 1.45) *p*=0.0001 |
| Model 2 Minimum SBP | 1.19 (1.06, 1.33) *p*=0.0001 |
| Model 3 Post-HD DBP | 1.26 (1.13, 1.40) *p*=0.0001 |
| Model 4 Minimum DBP | 1.16 (1.05, 1.30) *p*=0.0055 |

**Supplementary Table 2. Pressor approaches and risks of all-cause death in the matched dataset**

Values are given as HRs (95% CI). The Cox proportional hazards models were adjusted for blood pressures that showed a standardized difference of more than 10 % between the matched pressor and nonpressor groups.

Abbreviations: HR, hazard ratio; CI, confidence interval; HD, hemodialysis; SBP, systolic blood pressure; DBP, diastolic blood pressure.

**Supplementary Figure S1. Blood pressure and risk of all-cause death.**


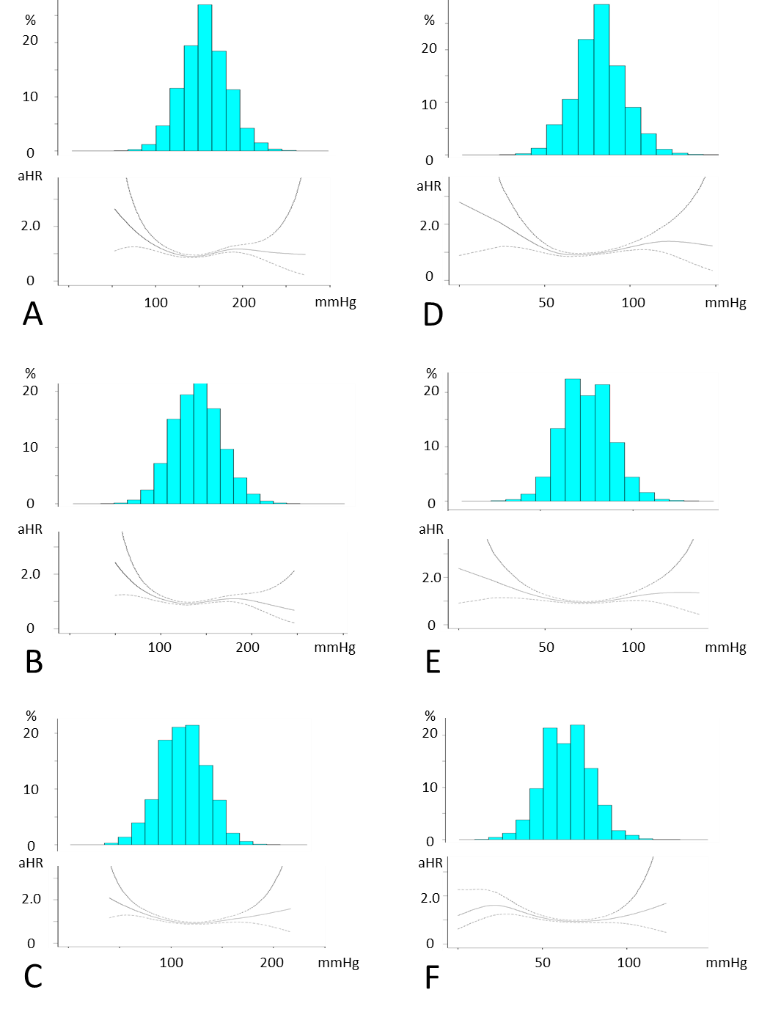


A, Pre-HD systolic blood pressure (*p*=0.0001)

B, Post-HD systolic blood pressure (*p*=0.0001)

C, Minimum systolic blood pressure (*p*=0.0001)

D, Pre-HD diastolic blood pressure (*p*=0.0001)

E, Post-HD diastolic blood pressure (*p*=0.0001)

F, Minimum diastolic blood pressure (*p*=0.0001)

The aHRs of all-cause death are shown with 95% CI (aHR, solid line; 95% CI, dashed line). The Cox proportional hazard models were adjusted for baseline characteristics such as gender, age, CVD, DM, ln(vintage), BMI, serum albumin, and creatinine levels, ln(CRP), hemoglobin level, fluid removal rate and blood pressures.

Abbreviations: aHR, hazard ratio adjusted for baseline characteristics; CI, confidence interval; HD, hemodialysis; SBP, systolic blood pressure; DBP, diastolic blood pressure.

**Supplementary Figure S2. Association between use of pressor approaches and risk of all-cause death in matched subjects.**


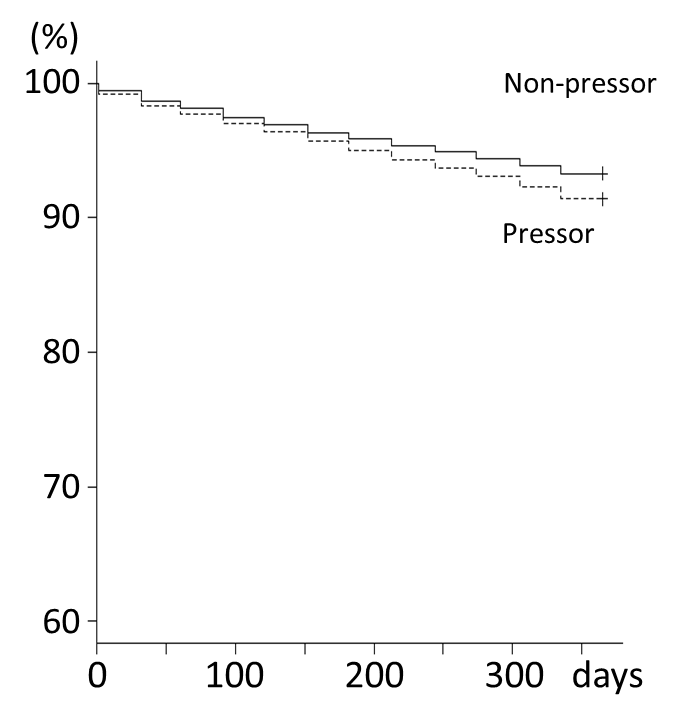


The Kaplan-Meier survival curve showed a lower survival probability in the matched pressor group than in the matched nonpressor group (Log-rank and Wilcoxon tests, *p*=0.0001).

Solid line is the matched nonpressor group.

Dashed line is the matched pressor group.

Abbreviations: pressor, matched pressor group; nonpressor, matched nonpressor group.
